# Non-invasive kinetic modelling of PET tracers with radiometabolites using a constrained simultaneous estimation method: evaluation with ^11^C-SB201745

**DOI:** 10.1186/s13550-018-0412-6

**Published:** 2018-07-03

**Authors:** Hasan Sari, Kjell Erlandsson, Lisbeth Marner, Ian Law, Henrik B.W. Larsson, Kris Thielemans, Sébastien Ourselin, Simon Arridge, David Atkinson, Brian F. Hutton

**Affiliations:** 1 Institute of Nuclear Medicine, L.5 University College Hospital, 235 Euston Road, London, NW1 2BU UK; 2 Neurobiology Research Unit, Center for Integrated Molecular Brain Imaging (CIMBI), Rigshospitalet, University of Copenhagen, Copenhagen, Denmark; 3 Department of Clinical Physiology, Nuclear Medicine and PET, Rigshospitalet, University of Copenhagen, Copenhagen, Denmark; 4 Centre for Medical Imaging Computing, Faculty of Engineering, University College London, London, UK; 5 Centre for Medical Imaging, Division of Medicine, University College London, London, UK; 6 Centre for Medical Radiation Physics, University of Wollongong, Wollongong, New South Wales, Australia

**Keywords:** Positron emission tomography, PET/MR, Kinetic modelling, Arterial input function

## Abstract

**Background:**

Kinetic analysis of dynamic PET data requires an accurate knowledge of available PET tracer concentration within blood plasma over time, known as the arterial input function (AIF). The gold standard method used to measure the AIF requires serial arterial blood sampling over the course of the PET scan, which is an invasive procedure and makes this method less practical in clinical settings. Traditional image-derived methods are limited to specific tracers and are not accurate if metabolites are present in the plasma.

**Results:**

In this work, we utilise an image-derived whole blood curve measurement to reduce the computational complexity of the simultaneous estimation method (SIME), which is capable of estimating the AIF directly from tissue time activity curves (TACs). This method was applied to data obtained from a serotonin receptor study (^11^C-SB207145) and estimated parameter results are compared to results obtained using the original SIME and gold standard AIFs derived from arterial samples. Reproducibility of the method was assessed using test-retest data. It was shown that the incorporation of image-derived information increased the accuracy of total volume of distribution (V _T_) estimates, averaged across all regions, by 40% and non-displaceable binding potential (BP _ND_) estimates by 16% compared to the original SIME. Particular improvements were observed in K_1_ parameter estimates. BP _ND_ estimates, based on the proposed method and the gold standard arterial sample-derived AIF, were not significantly different (*P*=0.7).

**Conclusions:**

The results of this work indicate that the proposed method with prior AIF information obtained from a partial volume corrected image-derived whole blood curve, and modelled parent fraction, has the potential to be used as an alternative non-invasive method to perform kinetic analysis of tracers with metabolite products.

## Background

Kinetic analysis methods are often used to extract quantitative measurements from positron emission tomography (PET) scans, such as cerebral blood flow, metabolism and receptor distribution. The majority of these methods require a precise knowledge of the arterial input function (AIF) to obtain the concentration of the available PET tracer in the blood plasma. However, measurement of the AIF can be a challenging task in dynamic PET studies, particularly when radioactive metabolites are present in the blood. In such cases, the fraction of the metabolite-free parent compound needs to be determined before performing kinetic analysis.

The gold standard method to measure the AIF is arterial blood sampling which involves serial blood sampling from a radial artery during the course of the scan. These samples are then analysed to determine the concentration of the PET tracer in blood plasma over time. In the presence of radiometabolites, additional arterial samples are often required to determine the metabolite and parent compound fractions. Although it is regarded as the most accurate method for the measurement of the AIF, arterial sampling is often avoided in practice due to its invasiveness.

Image-derived input function (IDIF) estimation is often regarded as a more practical alternative to arterial sampling, where the AIF is derived from reconstructed PET images [1–3]. IDIF methods suffer from the limited resolution of PET scanners which can cause partial volume (PV) effects. Some researchers propose the use of anatomical information from MR or CT images to correct for PV effects and improve the accuracy of derived IDIFs [4, 5]. However, IDIF methods alone are not able to separate the concentration of parent compound when radioactive metabolites are present in the blood. In such cases, several blood samples are still required to perform metabolite correction [1, 6].

An alternative AIF estimation method used in brain studies is the simultaneous estimation method (SIME) which is based on estimating the AIF by fitting multiple tissue time activity curves (TACs) derived from different brain regions [7, 8]. In this approach, the AIF is defined as a parametric model where its parameters are simultaneously estimated with kinetic rate constants for brain tissues. This method was initially proposed for use in ^18^F-FDG studies but was later shown to be applicable to other tracers [6]. Since the AIF is estimated purely computationally in SIME, this method can be particularly useful to derive the metabolite-free AIF non-invasively from the PET data. However, simultaneous estimation of AIF and kinetic rate constants from multiple regions involves estimation of a large number of parameters, resulting in bias and poor precision in the estimates [7]. Due to this problem, SIME is often complemented with several blood samples obtained at late time points to scale the estimated AIF. Furthermore, AIFs estimated using SIME tend to have accurate area under the curve but not necessarily the exact AIF shape, especially during the period following injection. Similar to conventional compartmental modelling, SIME can be more robust in deriving the macroparameters, such as volume of distribution (V _T_) and binding potentials (BP), rather than individual kinetic parameters [9]. This is due to macroparameters being a function of kinetic parameters which are correlated. Hence, errors on kinetic parameter estimates often cancel out in the calculation of macroparameters.

We have previously shown with simulated ^18^F-FDG data that the performance of the SIME method can be significantly improved if some prior information about the early part of the AIF can be included from another source, such as an image-based measurement or an MRI-derived AIF [10]. In other work, prior AIF bolus information was utilised in ^18^F-FDG studies and was shown to work well when complemented with late blood samples [11].

Another source of uncertainty in SIME is the presence of cerebral blood volume (CBV) in TACs. Although this volume is usually small in TACs extracted from brain regions, the contribution of the tracer concentration in the CBV to the total TAC might not be negligible and needs to be accounted for in the kinetic modelling. In the original SIME, the total tissue output of each region is modelled by summing the products of estimated parent AIF with the tissue and blood concentrations. This approach is not ideal since the CBV component is linked to whole blood concentration rather than the parent AIF and these concentrations can have substantial differences over the course of the scan.

In this article, we propose a constrained SIME method which utilises information from an image-derived whole blood curve [5] to reduce the complexity of the SIME. Our aim is to aid the SIME optimisation by using this image-based measurement to define the AIF peak and to differentiate whole blood activity from parent tracer activity in the plasma. Our ultimate goal is to develop a fully non-invasive method to perform kinetic analysis of PET tracers with metabolites. The method is evaluated in a study using ^11^C-SC207145 [12] which is a radioligand used in quantification of 5-HT4 receptors in serotonin studies investigating memory and learning. The performance of the constrained SIME was assessed by comparing the results to those obtained using the original SIME and the gold standard arterial blood sample-derived AIFs.

## Methods

### SIME

The SIME works on the assumption that the parent AIF is common to all of the TACs and can be expressed using a mathematical function. In its original implementation, Feng et al. used an input function model [13] which is a sum of a gamma variate function and two exponentials. This function has six parameters and can be written as Eq. 1.

1 $$ \mathrm{C_{\mathrm{{P}}}}(\mathrm{t})=(\mathrm{A_{1}}\mathrm{t}-\mathrm{A_{2}-A_{3}})e^{-\lambda_{1} \mathrm{t}}+\mathrm{A_{2}}e^{-\lambda_{2} \mathrm{t}}+\mathrm{A_{3}}e^{-\lambda_{3} \mathrm{t}}  $$

The six parameters of the AIF function are estimated together with the kinetic parameters by fitting the TACs for multiple brain regions simultaneously. Therefore, when a two-tissue compartment (2-TC) model with four rate constants (K_1_–k_4_) is used to model *m* number of regions of interest (ROIs), 4*m*+6 parameters are included in the optimisation. The cost function can be written as follows:

2 $$ \phi(p)=\sum\limits_{i=1}^{m}\sum\limits_{j=1}^{n} w_{i,j} \left[\bar{E_{i}}(\mathrm{t}_{j})-M_{i}(\mathrm{t}_{j}) \right]^{2}  $$

where $\bar {\mathrm {E}}_{i}$(t _*j*_) is the estimated model output, *M*_*i*_(t _*j*_) is the measured PET activity concentration at the *j*th time frame of the *i*th region. Symbol *m* represents the number of TACs to be fitted, *n* represents the number of time frames for each TAC and *w*_*i*,*j*_ are the weights applied to the model and were set to 1 in our implementation to apply uniform weighting. In order to account for the measured activity in a PET scan, $\bar {\mathrm {E}}_{i}$(t _*j*_) is computed by averaging *E*_*i*_ over the length of the scanning interval:

3 $$ \bar{E_{i}}(t_{j})=\frac{1}{\Delta t_{j}}\int_{t^{-}_{j}}^{t^{+}_{j}}E_{i}(s)ds  $$

4 $$ E_{i}(t)=(1-V_{b}) C_{\mathrm{{P}}}(t) \otimes h[K_{1},k_{2},k_{3},k_{4}]_{i} + V_{b} C_{\mathrm{{WB}}}(t)  $$

where $t_{j}^{-}=t_{j}-\Delta t_{j}/2$, $t_{j}^{+}=t_{j}+\Delta t_{j}/2$ and *Δt*_*j*_ is length of frame *j*. *C*_WB_ represents the whole blood TAC, *C*_P_ is the parent AIF, *h* is the impulse response of the 2-TC model and *V*_*b*_ is the cerebral blood volume. As the original SIME does not include any prior measurement of *C*_WB_, *C*_P_ was used to replace it in Eq. 4.

### Constrained SIME

Some tracers can be converted into metabolite side products, causing an IDIF directly derived from PET images to be incorrect as the contribution of radioactive metabolites cannot be determined. The current standard method to measure the concentration of metabolites includes the collection of multiple arterial samples during the course of the PET scan. The concentration of tracer at each sample is measured to determine the whole blood curve. Next, the concentration in blood plasma is measured and plasma to whole blood ratios are plotted at each sample time point. These samples are often fitted with a linear function to be used as the plasma to whole blood ratio.

During a standard metabolite correction procedure, the fraction of parent tracer to total activity in plasma, known as the parent fraction, is determined from the plasma samples. This parent fraction is then fitted using a parametric function, such as a bi-exponential function or the Hill function, and the fitted curve is used to convert the plasma curve to a metabolite-free AIF [14]. The Hill function can be described as follows:

5 $$ \mathrm{f_{Hill}}(\mathrm{t})=1-\frac{(1-a)\mathrm{t}^{b}}{c+\mathrm{t}^{b}}   $$

Hence, the parent tracer concentration, *C*_P_, can be expressed as a product of the whole blood curve, fitted Hill function and plasma to whole blood ratio as shown in Eq. 6.

6 $$ \mathrm{C_{P}}(\mathrm{t})=\mathrm{C_{WB}}(\mathrm{t}) \mathrm{f_{Hill}}(\mathrm{t}) \mathrm{C_{ratio}}(\mathrm{t})  $$

where C _ratio_ is the ratio of tracer concentration in plasma to whole blood as a function time and modelled using a straight line equation: *C*_*ratio*_=*d*×t+*e*.

**Fig. 1:**
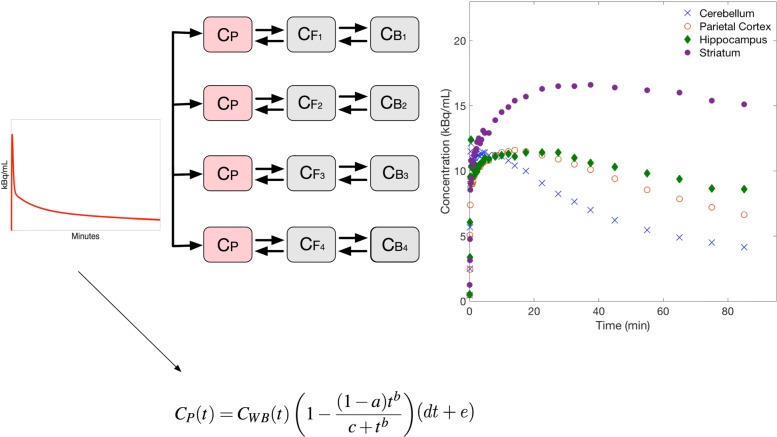
Constrained SIME model used in the analysis. *C*_P_ represents the AIF, *C*_F_ represents the free state and *C*_B_ of the 2-TC model. The *C*_P_ was modelled as a product of plasma to whole blood ratio and the Hill function. Three parameters of the Hill function (*a*, *b*, *c*) and two parameters of the straight line function (*d*, *e*) were optimised simultaneously by fitting *N* (where *N*=4) TACs extracted from different brain regions. *C*_WB_ is derived from PV-corrected PET images. TACs derived from the cerebellum, parietal cortex, hippocampus and striatum from one subject are shown on the right

If *C*_WB_ can be reliably measured from carotid arteries in partial volume-corrected PET images, the relationship shown in Eq. 6 can be used to model the AIF in the SIME cost function. In this implementation, the input function part of the cost function contains only five unknown parameters, three from the Hill function and two from the linear function used to model the plasma to whole blood ratio. The constrained SIME method is illustrated in Fig. 1.

### Data acquisition

All of the scans included in this study were performed at Copenhagen University Hospital, Rigshospitalet, Copenhagen, Denmark. The study followed the ethical guidelines from the Helsinki declaration of 1975 (revised 1983) and was approved by the Ethics Committee for Copenhagen and Frederiksberg ((KfF)01-274821). All subjects gave written informed consent. The data were collected as part of previous work published by Marner et al. [12] and have been included in the current study from the Cimbi Database [15] after approval from the Danish Data Protection Agency (2015-331-1254).

Six healthy volunteers (three males and three females, age range 21–44 years) were included in this study. Test and retest scans were performed on the same day with identical protocols. The PET data were acquired on an 18-ring GE Advance scanner (GE Healthcare, Milwaukee, WI, USA). Each subject received an intravenous administration of ^11^C-SC207145 with a mean activity of 572 MBq (range 512–601 MBq), and high specific activity (mean 48.4 GBq/ *μ*mol, range of 34.2–71.0 GBq/ *μ*mol). Dynamic PET acquisition was started before the tracer injection and 3D PET data were acquired for 2 h. 3D-filtered backprojection (6-mm Hann filter and 8.5-mm axial ramp filter) was used in the image reconstruction and the PET images were reconstructed on 128 × 128 × 35 volumes with a voxel size of 2.0 mm × 2.0 mm × 4.25 mm. A transmission source was used to derive maps for attenuation correction [16]. PET data were corrected for randoms, scatter and deadtime. Frames were binned using the following durations: 6 × 5 s, 10 × 15 s, 4 × 30 s, 5 × 2 min, 5 × 5 min and 5 × 10 min. As part of the PET study, arterial blood samples were collected from the radial artery at 46 time points with 5-s intervals in the early part of the scan to get a good definition of the bolus shape. Whole blood and plasma radioactivity were measured in a well counter (COBRA 5003, Packard Instruments, Meriden, CT, USA). Additionally, six arterial samples were withdrawn at times 3.5, 10, 17.5, 32.5, 55 and 85 mins to measure the concentration of metabolites. The parent fraction was measured using a column-switching HPLC method [17] which was previously shown to provide a reliable determination of radioactive products in plasma for ^11^C-labelled radiopharmaceuticals [18]. The Hill function was fitted to the measured parent fraction and was used to obtain the metabolite-free input function. The arterial samples were corrected to account for the delay between the radial artery and the brain by applying a negative time shift to match the first PET frame. No dispersion correction was performed.

Subjects also had structural MRI scans on a 3T Siemens Trio Scanner (Siemens Healthcare, Erlangen, Germany). Three-dimensional volumetric T1-weighted MPRAGE images (TR = 1380 ms, TI = 800 ms, TE = 2.6 ms and flip angle = 9°) of the head were acquired in sagittal planes for each subject. Images were reconstructed using a 256 × 256 × 256 matrix with voxel dimensions of 1 × 1 × 1 mm.

### Data analysis

An adapted version of an IDIF extraction method previously validated on ^18^F-FDG was used to derive the TAC of the tracer in the whole blood [5]. As higher resolution time-of-flight (TOF) MR angiograms were not collected as part of this study, MPRAGE images were used in the carotid artery segmentation. One of the six subjects had no visible arteries on the MPRAGE image and was excluded from the analysis as a reliable carotid artery segmentation was not feasible. For the rest of the subjects, carotid arteries were segmented using ITK-SNAP software application [19]. As part of the segmentation procedure, MPRAGE images were thresholded to retain the intensities greater than one third of the maximum image intensity. This step was used to separate the carotid arteries with higher signal from the background regions and replaced the automatic tissue classification method used in the originally published method. The tissue classification method did not yield the desired results for these datasets possibly due to the presence of high T_1_ signal in the background regions close to the arteries. Once the MPRAGE images were thresholded, a region-growing algorithm with active contour evolution was applied to delineate the arterial voxels [19].

The dynamic PET frames were registered to the MPRAGE images using a six-parameter rigid registration. The first 10 frames (90 s) of the dynamic PET data were added together to obtain a summed image with a high activity in the carotid arteries. This summed image was then used in the image registration which was performed using FSL Linear Image Registration Tool (FLIRT) [20]. The resulting transformation matrix was used to coalign all of the PET frames to the MPRAGE image. PET frames were resampled to MR space using tri-linear interpolation. The coregistered PET images were corrected for partial volume (PV) effects using the single-target correction (STC) method [5] where the segmented carotid artery mask was used as the only region of interest. An image-derived PSF of 6.8-mm full width half maximum (FWHM) was used in the PV correction. The mean intensity in the segmented artery region of each PV-corrected PET frame was calculated to derive the whole blood curve [5].

SIME requires TACs from multiple brain regions with different kinetic behaviours to estimate the AIF. In our implementation, TAC from the cerebellum was used to reflect a low binding region, TACs from the parietal cortex and hippocampus were used to reflect moderate binding regions and TACs from the striatum was used as a high binding region. Measured TACs from a single study are illustrated in Fig. 1. Segmentation of these regions was performed at Rigshospitalet, Copenhagen, Denmark [12]. The MPRAGE image of each subject was coregistered to the PET data and was parcellated into 19 different regions in the left and right hemispheres automatically using the Pvelab software package [21]. The segmented ROI masks were applied to the PET data to extract the desired TACs.

The SIME model was implemented in MATLAB (MathWorks, Inc., Natick, MA) using the COMKAT software package [22] which contains software libraries to build and solve compartmental models and perform parameter estimations. SIME optimisation was performed using the ordinary least squares method with uniform weighting applied to all time frames [23]. An analytical Jacobian of the AIF model was calculated to aid the optimisation. All four regions of interest were modelled using a two-tissue compartment model. The value of cerebral blood volume was not estimated and was assumed to be 5% for all regions. The *C*_WB_ was derived from PV-corrected PET images. Only the first 90 min of dynamic PET data were used in the analysis as this was previously shown to be acceptable for this tracer [12]. In the original SIME method, the six parameters of Feng’s AIF model [13] are estimated together with the kinetic parameters.

In the constrained SIME method, three parameters of the Hill function and two parameters of the plasma to whole blood ratio were estimated by fitting the tissue TACs simultaneously and the estimated parameters were used with Eq. 3 to derive the parent AIF. This AIF was then used to fit each TAC individually to obtain the kinetic parameters K_1_, k_2_, k_3_ and k_4_. This second TAC fitting step was applied to re-estimate the kinetic parameters of each region of interest in a reduced parameter space per region [8]. Total volume of distribution (V _T_) was computed for each region using Eq. 7 and non-displaceable binding potential (BP _ND_) was calculated using Eq. 8, with the cerebellum used as the reference region, V _ND_.

7 $$ \mathrm{V_{T}}=\frac{K_{1}}{k_{2}}\left(1+\frac{k_{3}}{k_{4}} \right)  $$

8 $$ \mathrm{BP_{ND}}=\frac{\mathrm{V_{T}}-\mathrm{V_{ND}}}{\mathrm{V_{ND}}}  $$

The results of the constrained SIME and original SIME were also compared to the gold standard method, where the metabolite-corrected AIF derived from arterial samples was used to derive the kinetic parameters, V _T_ and BP _ND_. Statistical comparisons were performed using paired *t* tests. The results obtained from test and retest studies were used to compare the reliability of V _T_ estimates obtained using the proposed method and arterial samples. The intraclass correlation coefficient (ICC) was computed using Eq. 9:

9 $$ \text{ICC}=\frac{\text{BMS}-\text{WMS}}{\text{BMS}+\text{WMS}}  $$

where BMS is the mean sum of squares between subjects and WMS is the mean sum of squares between the test and retest studies for each subject. This produces a score between − 1 and +  1 which represents minimum and maximum reliability respectively.

## Results

Throughout this section, the original SIME with no prior information is referred to as SIME _original_, the constrained method with prior image-derived information is referred to as SIME _constrained_ and the AIF derived from metabolite-corrected arterial samples is referred to as AIF _samples_.

**Fig. 2:**
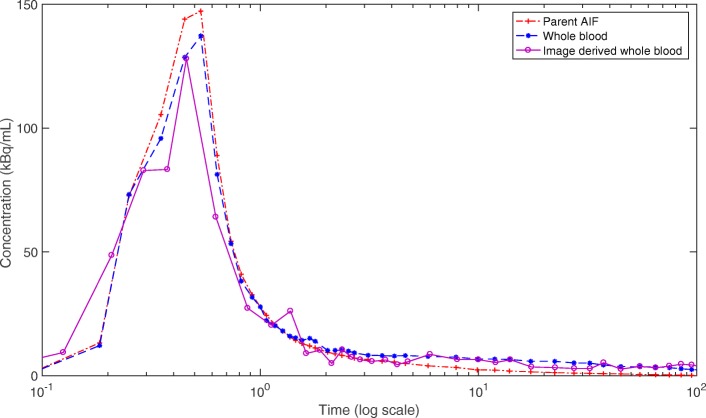
Comparison of image-derived and arterial blood sample-derived whole blood curves for one subject plotted together with the metabolite-free parent AIF derived from blood samples. The *x*-axis is set to log scale to display curve peaks and tails clearly

The accuracy of the image-derived whole blood TAC measurement was visually assessed against the blood sample-derived whole blood curves. Examples of these two curves are illustrated in Fig. 2 together with the AIF _samples_. A paired *t* test was used to compare the area under curves (AUCs) of the measured and image-derived whole blood curves. The peak and the tail parts of the curves were assessed separately. No significant difference was observed between the AUCs of the whole blood curve tails (*P*=0.10). However, a statistically significant difference (*P* < 0.05) was present between the image-derived and blood sample-derived whole blood peaks. A good agreement was observed in 9 of 10 subjects, but in one subject, the image-derived method yielded underestimated concentrations both in the peak and tail of the whole blood curve. The reason for this is unknown but may be caused by the errors propagated from segmentation and registration steps.

**Fig. 3:**
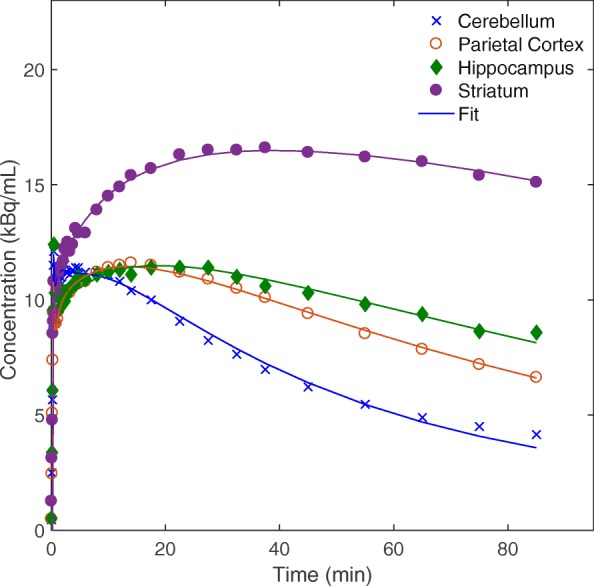
TACs from one subject with simultaneously fitted curves using SIME _constrained_ modelling

**Fig. 4:**
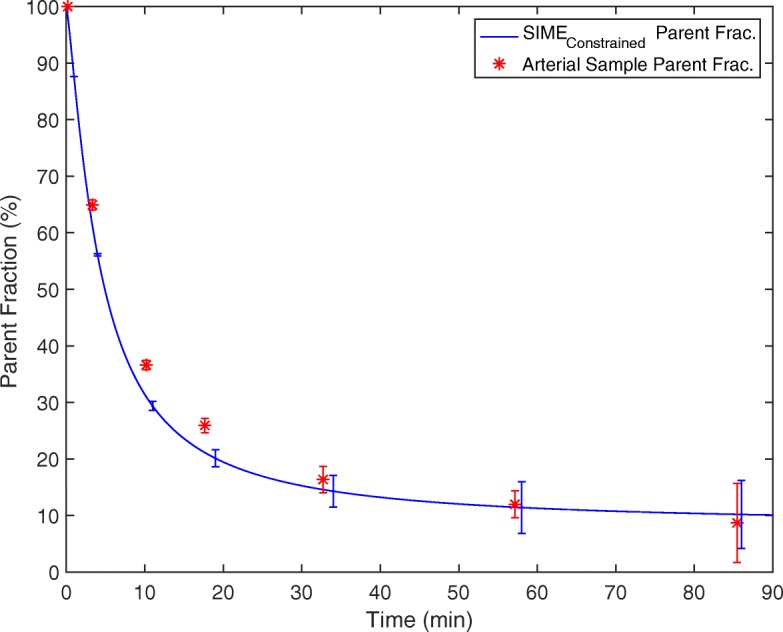
Average of the parent fractions estimated using the SIME _constrained_ plotted together with average of parent fractions derived from plasma samples. Error bars represent the variation between estimated and measured parent fraction curves from 10 datasets

During SIME _constrained_ optimisation, the parent fraction is estimated together with kinetic parameters by fitting the multiple tissue TACs simultaneously. Plots of simultaneously fitted TACs for a representative subject is shown in Fig. 3. The model provided good fits to the experimental data in all regions. Figure 4 illustrates the average of the estimated Hill functions using SIME _constrained_ together with blood sample-derived parent fractions averaged across 10 datasets. Error bars are included to show variability in fitted parent fractions at blood sample data points. A very good agreement was observed between the blood sample-derived parent fractions and SIME _constrained_ estimated parent fractions. Comparison of parent fraction values at metabolite sample time points did not show any significant difference between SIME _constrained_ and AIF _samples_ (*P*=0.58). These results indicate that SIME _constrained_ was able to estimate parent fractions with a comparable accuracy to blood samples. Plasma to whole blood ratio values at these time points were also compared and no significant difference was seen between the plasma to whole blood ratio curves estimated using SIME _constrained_ and derived from arterial samples (paired *t* test, *P*=0.28).

**Fig. 5:**
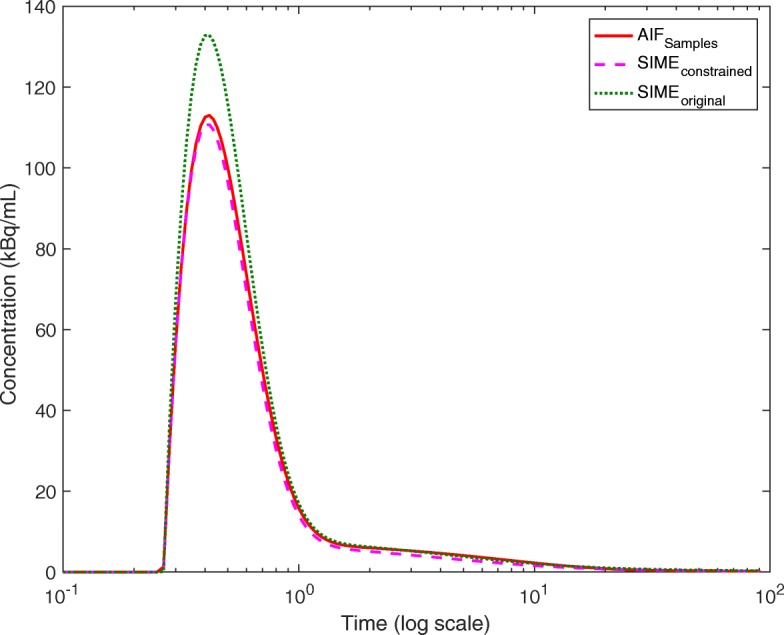
AIFs derived using SIME _original_, SIME _constrained_ and AIF _samples_ plotted together for one subject. The *x*-axis is set to log scale to display curve peaks and tails clearly

**Fig. 6:**
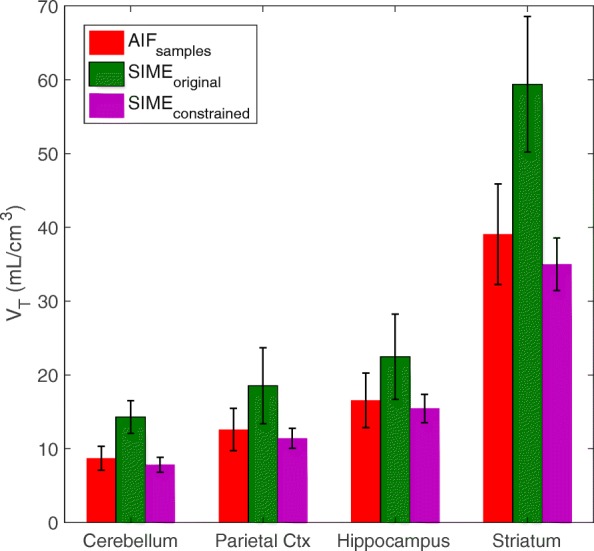
V _T_ estimated for four brain regions using the AIFs estimated from AIF _samples_, SIME _original_ and SIME _constrained_. The bars represent the V _T_ values estimated across 10 studies and error bars represent the standard deviation

AIFs estimated using the three methods are plotted in Fig. 5. The AIF estimated using SIME _constrained_ has a very good agreement with the gold standard AIF _samples_, particularly at the AIF peak whereas SIME _original_ overestimated the peak. AUCs of the peak and tails of the AIFs derived using AIF _samples_ and SIME _constrained_ were also compared using a pairwise *t* test and no statistically significant difference was observed (*P*=0.50 and *P*=0.52 respectively). The estimated AIFs were used to fit TACs derived from the cerebellum, parietal cortex, hippocampus and striatum and estimated kinetic parameters were used to calculate V _T_ in each region. The averaged results for 10 datasets are plotted in Fig. 6 together with results obtained using SIME _original_ and AIF _samples_. It can be seen that using SIME _original_ yielded V _T_ estimates with 65% error for the cerebellum, 47% for the parietal cortex, 36% for the hippocampus and 50% for the striatum. Using SIME _constrained_ reduced the averaged percentage error to − 10% for the cerebellum, − 10% for the parietal cortex, − 7% for the hippocampus and − 11% for the striatum. Averaged across all four regions, the absolute percentage error was reduced by 40% on V _T_ estimates and 16% in BP _ND_ estimates. SIME _constrained_ underestimated the V _T_ in all of the four regions whereas SIME produced overestimated V _T_ values.

**Fig. 7:**
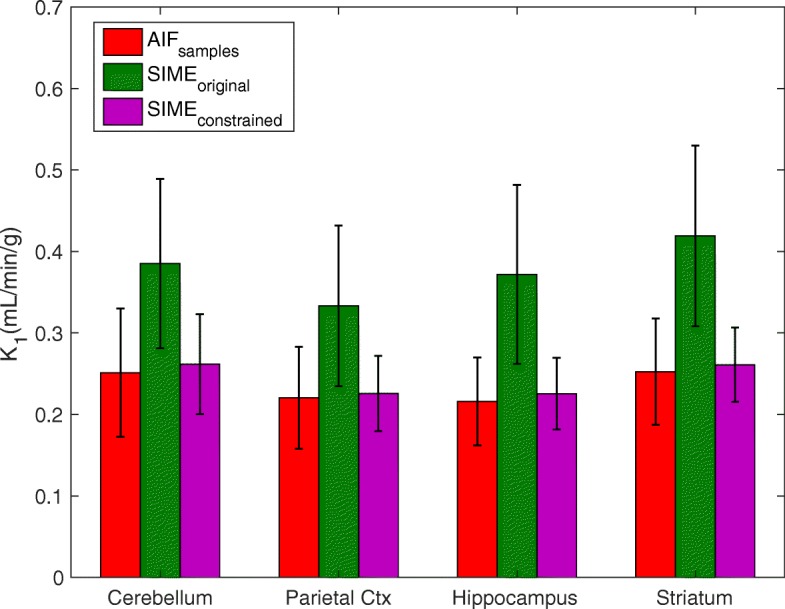
K_1_ estimated for four brain regions using the AIFs estimated from AIF _samples_, SIME _original_ and SIME _constrained_. The bars represent the K_1_ values estimated across 10 studies and error bars represent the standard deviation

The estimated K_1_ values are illustrated in Fig. 7. SIME _constrained_ resulted in a significant improvement in K_1_ estimates compared to SIME _original_. When the SIME _constrained_ was used, the percentage error dropped to 4% for the cerebellum and hippocampus, 2% for the parietal cortex and 3% for the striatum. Contrary to V _T_ results, SIME _constrained_ produced slightly overestimated K_1_ values whereas SIME _original_ significantly overestimated K_1_ in all regions. The difference between K_1_ estimates between SIME _constrained_ and AIF _samples_ was not statistically significant (*P*=0.08).

Non-displaceable binding potential estimates in Table 1 show that SIME _constrained_ was able to estimate this parameter with a reasonable accuracy whereas larger discrepancies were observed when SIME _original_ was used. SIME _original_ estimated BP _ND_ with 29.7, 30.3 and 8.6% error for the hippocampus, parietal cortex and striatum respectively whereas these errors were reduced to 10.0, 9.0 and 1.4% respectively with SIME _constrained_. However, results obtained using SIME _constrained_ had a larger standard deviation in BP _ND_ estimates by 7.0% on average compared to the results obtained by arterial samples.

**Table 1:** Average non-displaceable binding potential (BP _ND_) calculated across 10 scans with the standard deviation using input function derived from AIF _samples_, SIME _original_ and SIME _constrained_

	AIF _samples_	SIME _original_	SIME _constrained_
Cerebellum	–	–	–
Parietal ctx.	0.42±0.08	0.29±0.26	0.46±0.12
Hippocampus	0.90±0.22	0.63±0.60	0.98±0.23
Striatum	3.55±0.52	3.25±0.96	3.50±0.57

**Fig. 8:**
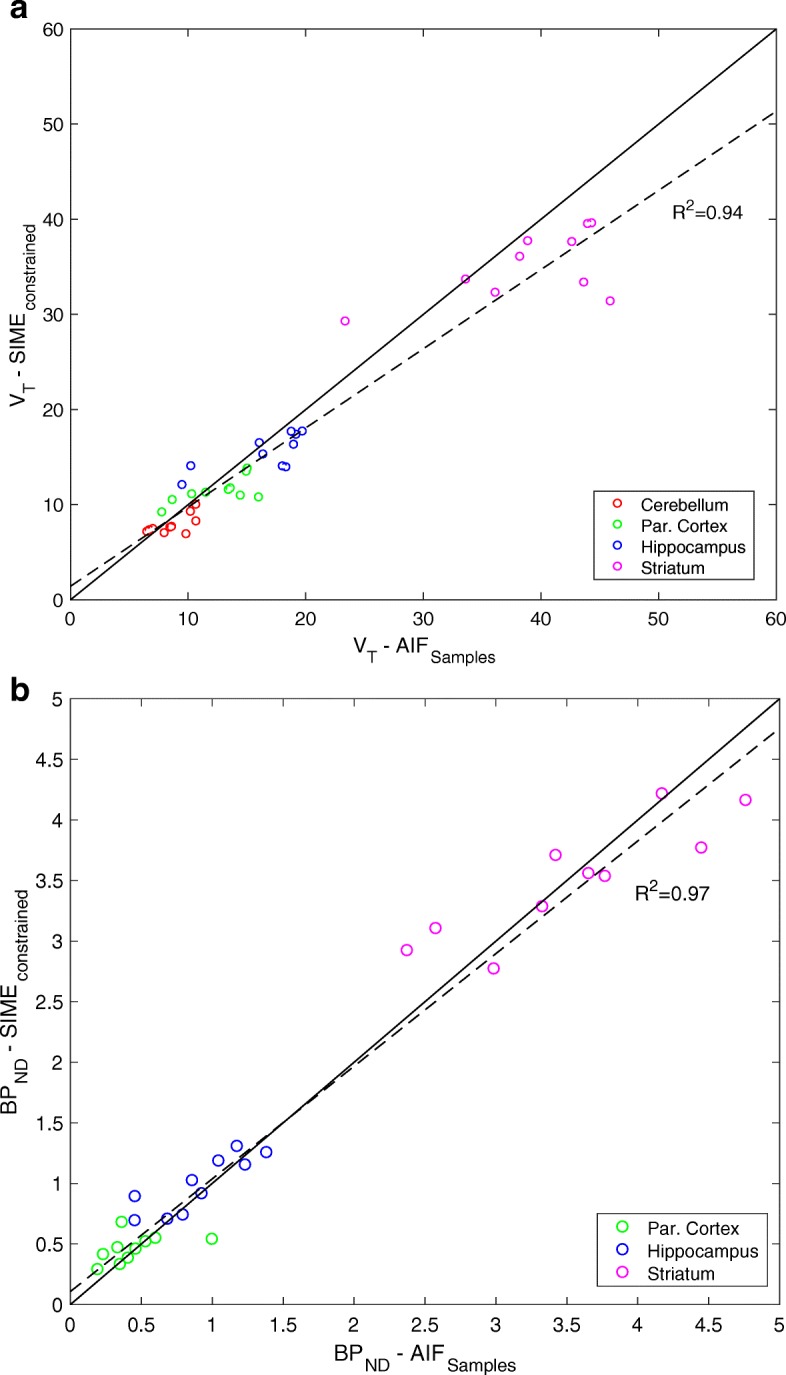
**a** V _T_ and **b** BP _ND_ estimated using SIME _constrained_ compared to V _T_ and BP _ND_ determined by AIF from arterial samples. Results from each scan are shown per each ROI. A linear function was fitted to the data points and plotted together with the line of identity

In Fig. 8a, V _T_ estimations obtained using the proposed method are plotted against the ones obtained using arterial samples. While a good agreement was seen between methods at lower V _T_ values, a deviation was seen as SIME _constrained_ underestimated V _T_ at higher values (i.e. in the striatum). Paired *t* test analysis showed statistically significant difference between estimated V _T_s from SIME _constrained_ and AIF _samples_ (*P*<0.05). Figure 8b shows that there was a very good relationship between the BP _ND_ between AIF _samples_ and SIME _constrained_. The linear fit to BP _ND_ estimates from all brain regions and scans shows that the SIME _constrained_ was able to derive matching results to the gold standard method, closely following the identity line. Paired *t* test showed no statistically significant difference (*P*=0.79) between BP _ND_ estimates using SIME _constrained_ and AIF _samples_.

**Fig. 9:**
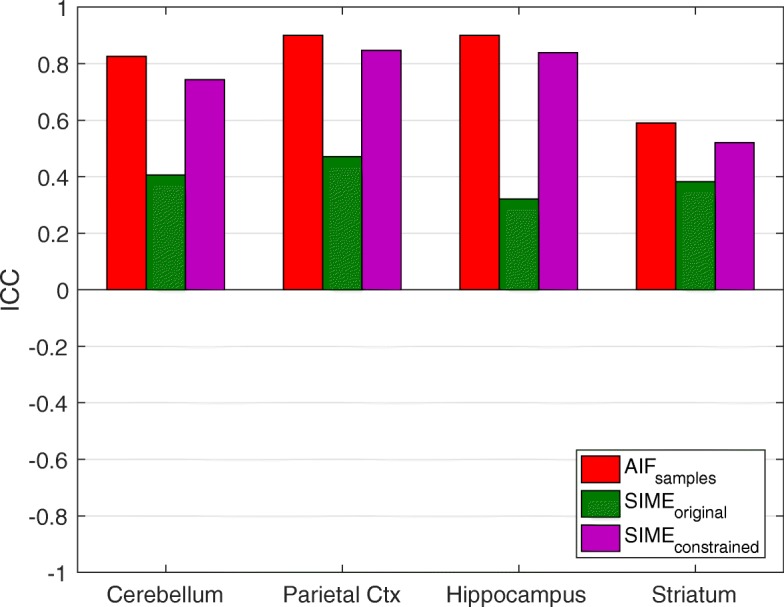
Intraclass correlation coefficient (ICC) scores of the V _T_ estimates obtained using AIF _samples_, SIME _original_ and SIME _constrained_. + 1 represents maximum reliability whereas − 1 represents minimum reliability

The intraclass correlation coefficient (ICC) results in Fig. 9 illustrate reproducibility of all three methods by comparing V _T_ estimates obtained from test and retest studies. AIF _samples_ yielded results with small test-retest differences and high ICC, whereas SIME _original_ produced positive but much lower ICC results in all regions. SIME _constrained_ produced reproducible results between test and retest scans, with ICC values of 0.74 for the cerebellum, 0.84 for the parietal cortex, 0.83 for the hippocampus and 0.52 for the striatum. These results indicate that SIME _constrained_ had only slightly lower reproducibility than the AIF _samples_.

## Discussion

In this paper, our work to incorporate image-derived whole blood concentration to improve the SIME analysis of dynamic PET data is presented. The proposed method is compared against the original SIME with no prior information and also against the gold standard arterial sample-derived AIF. Anatomical MR images were used with the STC method to correct for partial volume effects and to obtain an accurate estimation of the whole blood TAC from PET images. This whole blood curve was included in the SIME cost function to reduce the number of estimated parameters.

The constrained SIME was used to analyse dynamic PET data from ^11^C-SB201745 serotonin receptor studies. Kinetic analysis of this tracer requires information about concentration of metabolite products, which is traditionally extracted from multiple arterial samples. SIME can be considered as a non-invasive alternative to analyse such tracers but large errors were observed in the estimated parameters when no prior information is included to aid AIF estimation. Using information derived from MRI-based PV-corrected PET images improved the K_1_ and more importantly the V _T_ and BP _ND_ estimates which are the main macroparameters sought in the analysis of this tracer [12]. BP _ND_ estimates, based on the proposed method and the gold standard arterial sample-derived AIF, were not significantly different (*P*=0.7). The intraclass correlation results showed that the constrained SIME method was able to provide a reproducible performance in estimating V _T_ between test and retest studies. The original SIME showed a considerably poorer performance with lower ICC scores.

The significant improvement in K_1_ estimates can be explained by the direct link of this parameter to the early part of the AIF. The original SIME method has difficulty in accurately defining the AIF peaks but is more accurate in estimating the tail [6] especially when blood samples are used to anchor the estimated AIF. This makes it more successful in estimating macroparameters of kinetic analysis (i.e. K _i_, V _T_) rather than individual parameters. Since a reliable measurement of the whole blood curve is used in the constrained SIME, this method is superior in estimating the complete shape of the AIF curve, including the peak. This should be valid for most tracers except those with rapid uptake by red blood cells, which would result in differences between the plasma and whole blood curves.

The proposed method requires an accurate measurement of the whole blood curve from PET images, plasma to whole blood ratio and knowledge of an analytical function which can be used to define the parent fraction curve. Various models are available in the literature to model the parent fraction for different tracers [14]. In this work, the Hill function was used as it was previously shown that it is a suitable function to fit blood sample-derived parent fraction curves in ^11^C-SB201745 studies [12]. A constrained bi-exponential function, also with three parameters, is an alternative function that can be used in the analysis of this tracer, but this was not assessed in this paper. The plasma to whole blood ratio was parameterised as a straight line function where its parameters were optimised within the cost function.

In this work, the input function estimated from the constrained SIME method was used to fit individual TACs separately to estimate regional kinetic parameters. This individual TAC fitting step can be beneficial in avoiding local minima during the optimisation process, which is a risk when high number of parameters are estimated to fit multiple TACs at once. Furthermore, after estimating the AIF, it is possible to perform voxelwise kinetic analysis or fit some additional regions that were not included in the SIME procedure. In this scenario, it is best to re-fit the original SIME regions as well, so as to use the same procedure for all ROIs.

One limitation of this study was the arterial blood sampling protocol used to obtain the arterial input function. AIF was sampled in 5–10-s intervals for the first 2 min, which might affect the accuracy of the derived AIF peak. As the blood samples are obtained from the radial artery, which is distant to the brain, the measured AIFs can suffer from delay and dispersion effects. There are available methods in the literature to correct for these effects [24, 25] but errors can be present even after such corrections. Therefore, it can be argued that SIME has the potential to give more specific parameter estimates as it directly measures the AIF using information from TACs in the tissue of the interest.

The constrained SIME can be used to eliminate the need for arterial sampling which is conventionally performed in the analysis of PET tracers with radiometabolite products in the blood plasma. The accuracy of the image-derived whole blood curves depends on the performance of carotid artery segmentation, PET-MR image registration and the partial volume correction of PET images. The method used to obtain the image-derived whole blood measurements was previously validated using ^18^F-FDG datasets [5]. Results of the current study reconfirm that the blood concentration could reliably be measured from partial volume-corrected PET images. In order to use the proposed method, the study needs to include anatomical MR images to be used in carotid artery segmentation. TOF-MR angiography is capable of providing higher resolution images with a better contrast in the arteries and therefore more straightforward artery segmentation than the T1-weighted MPRAGE images used in this study.

In this study, proof of principle of using a constrained SIME method to non-invasively analyse PET tracers with radiometabolites was demonstrated. The proposed method may be applicable to other PET tracers provided that an accurate determination of the whole blood curve can be obtained and multiple time activity curves with different kinetic behaviours are included in the field of view.

## Conclusions

In this paper, we present a method which can be used to non-invasively analyse dynamic PET data in the presence of radiometabolite side products in the plasma. The proof of principle was demonstrated with ^11^C-SB201745 data. This method is intended as a general procedure although further evaluation is required for application to other tracers.

Anatomical MR images were utilised with the STC partial volume correction technique to derive reliable estimates of the whole blood TACs from coregistered PET images. The parent fraction of the tracer in the plasma and plasma to whole blood concentration relationship were modelled using parametric equations whose parameters were estimated within the SIME optimisation. The proposed method was able to derive K_1_ and macroparameters V _T_ and BP _ND_ with good accuracy and precision.
